# Predicting physiologically relevant SH3 domain mediated protein–protein interactions in yeast

**DOI:** 10.1093/bioinformatics/btw045

**Published:** 2016-02-09

**Authors:** Shobhit Jain, Gary D. Bader

**Affiliations:** ^1^Department of Computer Science and; ^2^The Donnelly Centre, University of Toronto, Toronto, ON, Canada

## Abstract

**Motivation:** Many intracellular signaling processes are mediated by interactions involving peptide recognition modules such as SH3 domains. These domains bind to small, linear protein sequence motifs which can be identified using high-throughput experimental screens such as phage display. Binding motif patterns can then be used to computationally predict protein interactions mediated by these domains. While many protein–protein interaction prediction methods exist, most do not work with peptide recognition module mediated interactions or do not consider many of the known constraints governing physiologically relevant interactions between two proteins.

**Results:** A novel method for predicting physiologically relevant SH3 domain-peptide mediated protein–protein interactions in *S. cerevisae* using phage display data is presented. Like some previous similar methods, this method uses position weight matrix models of protein linear motif preference for individual SH3 domains to scan the proteome for potential hits and then filters these hits using a range of evidence sources related to sequence-based and cellular constraints on protein interactions. The novelty of this approach is the large number of evidence sources used and the method of combination of sequence based and protein pair based evidence sources. By combining different peptide and protein features using multiple Bayesian models we are able to predict high confidence interactions with an overall accuracy of 0.97.

**Availability and implementation: Do**main-**Mo**tif Mediated Interaction **Pred**iction (DoMo-Pred) command line tool and all relevant datasets are available under GNU LGPL license for download from http://www.baderlab.org/Software/DoMo-Pred. The DoMo-Pred command line tool is implemented using Python 2.7 and C ++.

**Contact:** gary.bader@utoronto.ca

**Supplementary information:** Supplementary data are available at *Bioinformatics* online.

## 1 Introduction

Protein–protein interactions (PPIs) are physical associations between protein pairs in a specific biological context. Their knowledge provides important insights into the functioning of a cell. Previously, experimental detection of PPIs was limited to labor intensive techniques such as co-immunoprecipitation or affinity chromatography (Skrabanek **et al.**, 2008). Though the detected PPIs are largely accurate, these techniques are difficult to apply to whole proteome analysis. This led to the development of various high-throughput PPI detection protocols such as mass-spectrometry combined with affinity-purification, yeast two-hybrid and next-generation sequencing to detect PPIs at whole genome level (Braun *et al.*, 2013; Davy *et al.*, 2001; Ito *et al.*, 2001; McCraith *et al.*, 2000; Rain *et al.*, 2001; Uetz *et al.*, 2000; Yu *et al.*, 2011). However, genome-scale methods are also highly resource intensive and single projects and techniques do not cover all known protein interactions. Further, they only cover interactions in one organism at a time. Computational approaches designed to predict reliable and novel PPIs based on experimental interaction datasets have the advantages that they are inexpensive to apply to genomes, including those that are infeasible to tackle experimentally and this motivates their further development (Skrabanek *et al.*, 2008).

Multiple kinds of protein–protein interactions exist. We focus on interactions involving peptide recognition modules (PRMs), in particular Src homology three (SH3), which are important in many cellular signaling processes. These domains bind to small, linear sequence motifs (peptides) within proteins (Pawson and Nash, 2003). SH3 domains are approximately 60 amino acids long with five beta strands organized into two perpendicular beta sheets interrupted by a 3–10 helix (Pawson and Gish, 1992). They often bind to proline-rich regions and multiple classes have been recognized based on their binding motifs. Class I SH3 domains bind to [R/K]xxPxxP and class II bind to PxxPx[R/K] motifs (Mayer, 2001). They can also bind to proline-free regions containing arginine or lysine (Tong *et al.*, 2002). SH3 domains are involved in many regulatory or signaling processes, including endocytosis (Tonikian *et al.*, 2009), actin cytoskeleton regulation (Pawson and Schlessingert, 1993) and tyrosine kinase pathways (Schlessinger, 1994). Experimental methods such as phage display (Tong *et al.*, 2002; Tonikian *et al.*, 2008, 2009) and peptide microarray (Hu *et al.*, 2004; MacBeath and Schreiber, 2000; Stiffler *et al.*, 2007) have been used to identify the peptides binding to PRMs.

The computational problem under focus in this work is to use the SH3 domain binding peptides identified from phage display experiments to predict SH3 domain mediated PPIs in *S. cerevisiae*. A straightforward approach is to construct position weight matrices (PWMs) from phage peptides and scan the whole proteome for potential binding sites in target proteins using some threshold score (Obenauer *et al.*, 2003). The problem with this simple approach is the lack of contextual information, for example, the predicted binding site might not be accessible or it might lie within a structured part of protein (e.g. domain). Tonikian *et al.* (2009) addressed this problem by combining in vitro (phage display, peptide array screening) and in vivo (yeast two-hybrid) data to predict SH3 domain mediated PPIs in yeast. Verifying interactions using multiple experimental techniques improves the PPI confidence but it is both time and resource consuming. Lam *et al.* (2010) combined comparative and structural genomic features with PWMs to reduce the number of false binding sites. But they did not consider that PPIs are influenced by many cellular constraints including that interacting proteins must be in close proximity and should be part of same process. Peptide-only features are not sufficient for predicting high confidence physiologically relevant PRM mediated PPIs with binding site resolution. Jansen *et al.* (2003), Rhodes *et al.* (2005), Li *et al.* (2008), Zhang *et al.* (2012) and others considered multiple types of cellular constraints and combined different evidence sources for PPI prediction, but their approaches are designed for full length proteins and cannot be used to predict PRM mediated PPIs, including identification of binding sites. More recently, Chen *et al.* (2015) combined limited number of peptide and protein features for predicting PRM mediated PPIs in humans. Their protein features are based on one of the earlier the works in the field of ensemble PPI prediction (Jansen *et al.*, 2003). Since then many advances have been made in improving the performance of individual features in PPI prediction (Reimand *et al.*, 2012). Also, their method is not compatible with high-throughput binding peptide data, such as from phage display. Here, we make use of a larger set of evidence sources to predict SH3-mediated PPIs and their binding sites than has been collected previously and combine peptide level and protein level features in a single predictor.

## 2 Approach

**Fig. 1.**
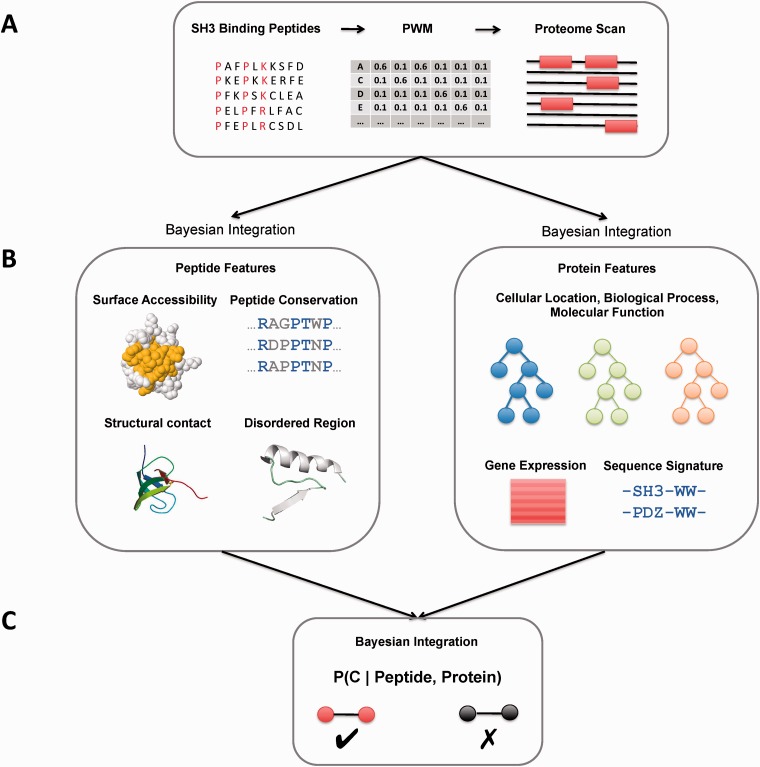
Work flow of PRM mediated PPI prediction pipeline. (A) Proteome is scanned using a PWM built with experimentally derived binding peptides (e.g. from phage display) of a given SH3 domain for potential interactors. (B) Separate Bayesian classifiers for peptide and protein features. (C) Integration of classifiers for predicting interacting and non-interacting protein pairs (Color version of this figure is available at Bioinformatics online.)

PRM mediated PPIs do not occur in isolation in the cell. They are influenced by different sequence-based and cellular constraints. For example, SH3 domains can only bind surface accessible regions, interacting proteins must be present in same cellular compartment, and proteins in the same biological process with correlated gene expression profiles are more likely to interact compared to randomly selected protein pairs. Thus, diverse types of information can be used to help predict physiologically relevant protein interactions. In our method, PWMs constructed using peptides from phage display experiments are used to scan the yeast proteome for potential targets. Peptide features: disorder, surface accessibility, peptide conservation and structural contact are combined using naïve Bayesian integration to score the PWM targets. Another naïve Bayesian model is used to combine protein features: cellular location, biological process, molecular function, gene expression and sequence signature to score the same targets. Scores from both peptide and protein classifiers are then combined using Bayes theorem to predict physiologically relevant SH3 domain mediated PPIs in yeast. Figure 1 shows the work flow of our PRM mediated PPI prediction pipeline.

## 3 Methods

### 3.1 Position weight matrix and proteome scanning

Position weight matrices (PWMs) are statistical models for representing sequence motifs. They are real valued *m *×* n* matrices, where *m* is the number of amino acids and *n* is the motif length. They are constructed using peptides from phage display experiments and then used to scan a protein sequences to find motif matches above a certain *P*-value threshold (Pizzi *et al.*, 2011; Wu *et al.*, 2000). Also, significant positions within the PWMs are identified and used in scoring peptide features: disordered region, surface accessibility and peptide conservation (see supplementary material for details).

### 3.2 Peptide features

#### 3.2.1 Disordered region

PRMs bind to small peptide stretches containing a specific motif. Specifically interactions between proteins having SH3 domains and their targets are often mediated by proline rich peptide sequences containing PXXP, [R/K]xxPxxP, PxxPx[R/K] motifs. Proline disrupts the secondary structure of a protein by inhibiting the formation of helices and sheets (Morgan and Rubenstein, 2013). Also, small linear motifs tend to accumulate in disordered regions of protein (Beltrao and Serrano, 2005; Davey *et al.*, 2010; Linding *et al.*, 2003). Beltrao and Serrano showed that the binding sites of SH3 domains in *S. cerevisiae* often lie within the disordered regions of a protein (Beltrao and Serrano, 2005). DISOPRED, a neural network based tool, is used to estimate the probability of the protein region being disordered.

(1) $$\text{DR}=\frac{\sum_{i}p_{i}=\{\begin{array}{cc} 1 & \text{if amino acid }i\text{ is disordered}\\ 0 & \text{otherwise} \end{array}}{N}$$

where *p_i_* is the disorder score of the *i*^th^ significant amino acid (either one for disordered or zero for ordered) and *N* is the number of significant amino acids in the binding site.

#### 3.2.2 Surface accessibility

Sequences present on a protein’s surface are more accessible to binding by SH3 domains than those that are buried inside a protein structure. The degree of solvent-accessible surface area of amino acid residues in a sequence indicates its level of exposure and is measured in terms of relative solvent accessibility (RSA) (Adamczak *et al.*, 2004; Lam *et al.*, 2010). We use SABLE (Adamczak *et al.*, 2004) to predict RSA values for target sequences. It uses a neural network based nonlinear regression model for continuous approximation of RSA values. Amino acid residues with RSA value ≥25% are considered to be exposed and available for binding (Adamczak *et al.*, 2004).

(2) $$\text{SA}=\frac{\sum_{i}p_{i}=\{\begin{array}{cc} 1 & \text{if }\mathrm{RSA}>=25\%\\ 0 & \text{otherwise} \end{array}}{N}$$

where *p_i_* is the surface accessibility score of *i*^th^ significant amino acid and *N* is the number of significant amino acids in the binding site.

#### 3.2.3 Peptide conservation

Biologically relevant peptides binding to yeast SH3 domains are more likely to be conserved in other yeast species (Beltrao and Serrano, 2005; Davey *et al.*, 2010). For measuring the conservation, orthologs of *S. cerevisiae* protein sequences in *C. glabrata, D. hansenii, K. lactis, Y. lipolytica, C. albicans, N. crassa* and *S. pombe* (an optimal set as selected by (Beltrao and Serrano, 2005)) are identified using INPARANOID (Remm *et al.*, 2001). The orthologous sequences are then aligned with MAFFT (Katoh *et al.*, 2002) and the unweighted sum-of-pairs method from AL2CO (Pei and Grishin, 2001) is used to estimate the conservation score of each position in the multiple sequence alignment (Lam *et al.*, 2010).

(3) $$\text{PC}=\frac{\sum_{i}p_{i}}{N}$$

where *p_i_* is the conservation score of the *i*^th^ significant amino acid and *N* is the number of significant amino acids in the binding site.

#### 3.2.4 Structural contact

Known 3-D structures of SH3 domains complexed with peptides can be used to assess the binding potential of a query SH3 domain and peptide by reducing residue-residue contacts in 3-D structures to a binary 2-D contact matrix (Chen *et al.*, 2008; Hui and Bader, 2010). Six yeast SH3-peptide co-complex PDB structures (1N5Z, 1SSH, 1ZUK, 2KYM, 2RQW, 2VKN) are used as base models. The Contact Map Analysis (CMA) tool from the SPACE software suite (Sobolev *et al.*, 2005) is used to reduce the 3-D structures to 2-D contact maps with residue level contact area for all base models. Query domain and peptide sequences are aligned with all base models using the Needleman–Wunsch algorithm and BLOSUM 62 substitution matrix to calculate the contact distance between aligned residues.

(4) $$\text{SC}=\underset{j}{\max\;}\frac{\sum_{i}c_{ij}}{N}$$

where *c_ij_* is the normalized contact area of the *i*^th^ aligned domain and peptide residues of the *j*^th^ base model. Alignment gaps in contact residues will negatively impact the average contact area as only the aligned residues are used for scoring (a gap at a position associated with a large residue contact area will reduce the SC score more than a gap associated with a smaller residue contact area). *N* is the number of aligned contact residues.

### 3.3 Protein features

#### 3.3.1 Cellular location, biological process, molecular function

Physical PPIs require proteins to be in close proximity to each other i.e. they should co-localize in the same cellular compartment. Also, interacting proteins are more likely to be part of same biological process or have the same function. The Gene Ontology (GO) contains a hierarchy of controlled terms describing cellular location, biological process and molecular function of proteins (The Gene Ontology Consortium, 2000). The functional relationship between two proteins can be quantified using GO. Semantic similarity can be used to quantify relationships between different GO terms in an ontology. The higher the semantic similarity score between GO terms annotated to two proteins, more likely that they will interact with each other (Jain and Bader, 2010). Topological Clustering Semantic Similarity (TCSS) (Jain and Bader, 2010) is an accurate semantic similarity measure for PPI prediction. It normalizes the GO hierarchy before computing semantic similarity, according to cutoffs defined in the original TCSS paper.

(5) CC=TCSS(a,b,ontology=C,cutoff=2.4)

(6) BP=TCSS(a,b,ontology=P,cutoff=3.5)

(7) MF=TCSS(a,b,ontology=F,cutoff=3.3)

where *a* and *b* are the query proteins and *C*, *P*, *F* are the cellular component, biological process and molecular function ontologies.

#### 3.3.2 Gene expression

Gene expression as a measure for assessing the confidence and biological relevance of high-throughput PPIs is based on the notion that the cell is optimized to co-express genes if they function together and if they function together, they are more likely to physically interact than by chance (Bhardwaj and Lu, 2005; Ge *et al.*, 2001; Grigoriev, 2001; Jansen *et al.*, 2002). Most PPI prediction methods that make use of gene expression profile (GEP) correlation with PPIs to predict novel interactions (Li *et al.*, 2008; Rhodes *et al.*, 2005) rely on observations from a single expression dataset which can lead to many false positives and true negatives, as not all genes are expressed under a particular set of experimental conditions. Using multiple GEPs clearly improves the performance of a predictor as shown in Supplementary Figure S1. Correlation coefficients from 86 gene expression profiles from GeneMANIA (Warde-Farley *et al.*, 2010) for a given pair of genes are combined using Fisher’s z transformation (Faller, 1981; Jain and Bader, 2010)

(8) $$\text{EX}=1-\frac{e^{2\bar{z}}+1}{e^{2\bar{z}}-1}$$

(9) $$\bar{z}=N^{-1}\sum_{i=1}^{N}\frac{1}{2}\text{ln}\left(\frac{1+r_{i}}{1-r_{i}}\right)$$

where *N* is the number of profiles and *r_i_* is the Pearson correlation of the *i*^th^ profile.

#### 3.3.3 Sequence signature

Sequence signature based PPI prediction methods are based on the notion that protein domains are correlated with specific functions. For instance, it has been shown that functionally related proteins have similar domain composition or they belong to the same ‘domain club’ (Jin *et al.*, 2009). Information content of co-occurring InterPro (Apweiler *et al.*, 2001) signatures extracted from sequences of an experimentally verified set of 22 707 PPIs from DIP (Salwinski *et al.*, 2004) is used to score novel interactions, as described by Sprinzak and Margalit (2001).

(10) $$\text{SS}=\sum_{ij}-{\text{log}}_{2}\left(\frac{p_{ij}}{p_{i}p_{j}}\right)$$

where *p_ij_* is the probability of seeing motif *i* on one protein and motif *j* on other protein in the experimentally verified PPI set, *p_i_* is the probability of seeing motif *i* and *p_j_* is the probability of seeing motif *j* in the same set.

### 3.4 Bayesian integration

The objective of a Bayesian PPI prediction model is to estimate the probability that a given protein pair interacts, conditioned on the biological evidence in support of that interaction. A naïve Bayesian model simplifies this problem by assuming independence between different types of biological evidence. While modeling the PRM mediated PPI prediction problem a set of observations are made on domain-peptides while others are made on full-length proteins. Assuming that peptide and protein features are independent of each other, two separate naïve Bayes models *M*_pep_ for peptide features and *M*_pro_ for protein features are built to independently assess the class probability *Y*. The posterior probabilities $P(Y|M_{\text{pep}})$ and $P(Y|M_{\text{pro}})$ are combined using Bayes’ theorem (Mitchell, 1997) (see supplementary material for details).

## 4 Results

### 4.1 Model training

The goal is to construct a generalized model which can predict high confidence, in vivo yeast SH3 domain–peptide physical interactions. To achieve this, both peptide and protein classifiers are trained on their respective positive and negative datasets. The peptide classifier is trained on a high confidence set of 628 SH3 domain–peptide interactions in yeast from the MINT database **(P1)** and an equal number of randomly selected negative interactions **(N1)**. The protein classifier is trained on a high confidence set of 5215 pairwise yeast PPIs from the iRefIndex database **(P2)** and an equal number of randomly selected negative interactions **(N2)** (see supplementary material for details).

### 4.2 Feature selection

**Fig. 2.**
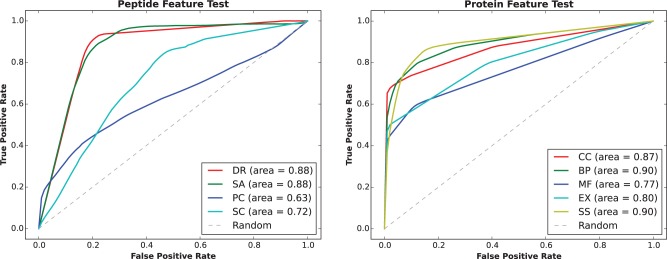
Prediction efficacy of individual peptide features: disordered region (DR), surface accessibility (SA), peptide conservation (PC), structural contact (SC); and protein features: cellular component (CC), biological process (BP), molecular function (MF), gene expression (EX), sequence signature (SS) (Color version of this figure is available at Bioinformatics online.)

Figure 2 shows the discriminatory power of individual features for peptide and protein classifiers. Disordered region (DR) and surface accessibility (SA) perform much better in separating positives from negatives as compared to structural contact (SC) and peptide conservation (PC). Prediction efficacy of PC is least among the peptide features. This is due to the difficulty distinguishing positive and negative interactions because both of these sets have high conservation scores caused by the high similarity of protein sequences (and peptides they contain) in general across different yeast species (Supplementary Fig. S2). Biological process (BP), cellular component (CC) and sequence signature (SS) outperform molecular function (MF) and gene expression (EX) in the protein feature set. Proteins could have the same molecular function but still belong to different processes and this could be one of the reasons behind molecular function feature’s weak performance. Gene expression data alone is not as powerful as others in discriminating positives from negatives (Kim *et al.*, 2014), which may be due to its moderate correlation with protein expression (i.e. gene expression may not imply that a functioning protein will be available for interaction) (Vogel and Marcotte, 2012).

Highly correlated features can negatively affect the performance of a naïve Bayesian classifier. Maximal information coefficient (MIC) is used to quantify the correlation between different features. DR and SA in the peptide feature set and CC and BP in the protein feature set are correlated with MICs of 0.72 and 0.5 respectively. The effect of correlation on classifier performance is measured by comparing different models without one of the correlated features. Further, to identify the feature subset which maximizes the performance of both classifiers, all possible combinations of features are compared using different statistical measures, such as area under ROC curve (AUROC), area under precision-recall curve (AUPRC), Brier score (BRIER), *F*_1_-score, Matthews correlation coefficient (MCC) and accuracy (ACC). Peptide and protein classifiers with all features outperformed other models on at least one of statistical measure (see supplementary material for details).

### 4.3 Model evaluation

Blind validation protocols are used to assess the predictive power of peptide *M*_pep_ and protein *M*_pro_ naïve Bayesian classifiers. The majority of interactions in the P1 dataset are from two peptide array experiments (Landgraf *et al.*, 2004; Tonikian *et al.*, 2009). This could lead to an experimental bias therefore, for blind testing, the peptide classifier is trained using interactions only from peptide array experiments and tested using interactions from all other experiments (no overlap between training and test datasets). Similarly, to make an unbiased assessment, the protein classifier was trained using P2 dataset but tested using the 2304 interactions (with no missing information) from the core subset of Database of Interacting Proteins (DIP) (Salwinski *et al.*, 2004) that do not overlap the P2 training set and are based on different filtering criteria compared to the MINT-inspired score used to select the iRefIndex P2 training set. The DIP core database includes PPIs derived from both small-scale and large-scale experiments that have been scored by quality of experimental methods, occurrence of interaction between paralogs (PVM), probable domain–domain interactions between protein pairs (DPV), and comparison with expression profiles (EPR) (Salwinski *et al.*, 2004). In a real world prediction scenario, both classifiers are expected to encounter cases with missing information. Therefore, the performance of both classifiers is also tested using an unfiltered blind set. The results are summarized in Table 1. The AUROC for peptide clasifier is 0.92 and ACC lies within the range [0.86, 0.87]. The protein classifier has an AUROC within the range [0.92, 0.94] and ACC is between [0.80, 0.83].

**Table 1.** Evaluation of peptide and protein classifiers

Test	Classifier	MCC	ACC	*F*_1_-score	AUROC
Filtered	Peptide	0.74	0.87	0.87	0.92
	Protein	0.68	0.83	0.83	0.94
Unfiltered	Peptide	0.72	0.86	0.86	0.92
	Protein	0.63	0.80	0.80	0.92

Matthews correlation coefficient (MCC), accuracy (ACC), *F*_1_-score and area under ROC curve (AUROC) of protein and peptide classifiers for blind tests are shown. MCC, ACC and *F*_1_-score are reported at threshold score ≥0.9. The filtered set has no missing values for any of the features, whereas unfiltered includes all feature data (as would be the case in a real world prediction scenario).

**Fig. 3.**
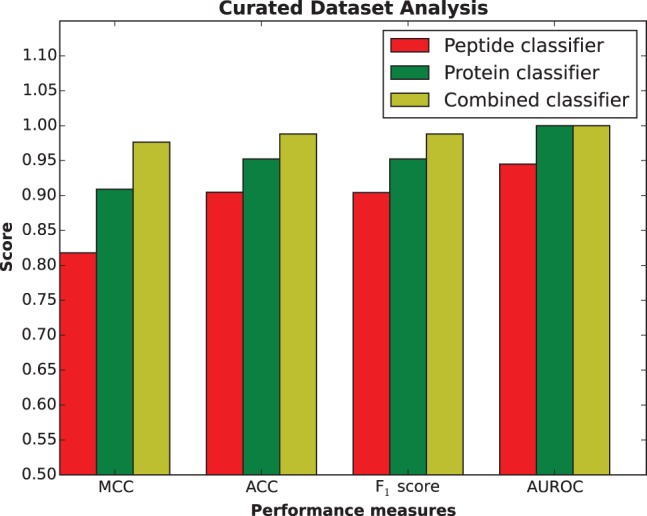
Performance of peptide, protein and combined classifiers on the curated SH3 domain mediated PPI set (Color version of this figure is available at Bioinformatics online.)

The efficacy of the combined peptide and protein model was tested on the manually curated SH3 domain mediated PPI set from Tonikian *et al.* (2009). Tonikian and co-workers curated interactions supported by multiple experiments through an exhaustive literature search. Not all interactions (especially those identified using two hybrid and overlay assays) in this set are mapped to the peptide sequence within the interacting partner (Tonikian *et al.*, 2009). Therefore, these sequences are scanned using the three P1 training set PWMs to identify binding sites and significant amino acid positions within those sites. Peptide and protein classifiers are trained on P1 and N1 (no overlap with curated set) and P2 and N2 datasets, respectively. A randomized negative test set is created in the same way as N1. Results from different statistical measures are summarized in Figure 3. The combined classifier outperforms both the peptide and protein classifiers on the curated set.

### 4.4 SH3 domain mediated PPI predictions

30 PWMs representing multiple binding specificities of 25 SH3 domains in yeast are constructed using phage display data from Tonikian *et al.* (2009) as described in Section 3.1 (Supplementary Tables S1 and S2). These PWMs are then used to predict SH3 domain–peptide interactions using the combined classifier. 534 unique PPIs (1481 binding sites) are predicted as positives for the stringent *P*-value PWM threshold of 1e-05 with no missing features (Supplementary Table S3). Approximately 55% (295 PPIs, 1139 binding sites) of these interactions are known at the PPI level (iRefIndex and MINT) and at least 172 (464 binding sites) out of 295 PPIs are known SH3 domain mediated interactions at the peptide level (with ≥60% overlapping binding site). For example, the FUS1p SH3 domain is known to bind the STE5p protein (verified by two-hybrid assay and phage display) via an R(S/T)(S/T)SL motif, supported by two separate studies (Kim *et al.*, 2008; Nelson *et al.*, 2004). This interaction is part of the predicted set. 143 (203 binding sites) out of 239 (342 binding sites) novel interactions are of high confidence with the combined classifier scores ≥0.9. Biological pathway enrichment (KEGG (Kanehisa, 2002) and Reactome (Croft *et al.*, 2014)) of the interactors reveal that a number of over-represented processes or pathways are associated with known SH3 domain biology such as endocytosis (Tonikian *et al.*, 2009; Xin *et al.*, 2013), MAPK signaling (Lyons *et al.*, 1996) and Rho GTPase signaling (Bishop and Hall, 2000) (Supplementary Table S4). For example, some interacting partners of the MYO3 SH3 domain are found to be enriched in PI3K/AKT signaling. AKT is known to regulate actin organization and cell motility during endocytosis (Enomoto *et al.*, 2005; Koral *et al.*, 2014). MYO3 is also implicated in actin organization for the internalization step in endocytosis (Toret and Drubin, 2006) (Supplementary Table S5). These examples support our results and suggest that our predicted interactions are biologically relevant.

## 5 Conclusion

We developed a novel method for predicting physiologically relevant PPIs in yeast. This method combines diverse binding site (peptide) features, including presence in a disordered region of the protein, surface accessibility, conservation across different yeast species, and structural contact with the SH3 domain, as well as protein features such as cellular proximity, shared biological process, similar molecular function, correlated gene expression and sequence signature. Two separate Bayesian models are used to combine peptide and protein features. Their respective posterior probabilities are further combined using Bayes rule for predicting high confidence interactions. The combination of peptide and protein models achieved a higher accuracy of 0.97 compared to individual models on a curated benchmark dataset from Tonikian *et al.* (2009). Disordered region and surface accessibility data from the peptide feature set and biological process, cellular location and sequence signature information from the protein feature set are able to separate positive from negative interactions significantly better than other features. The method presented is generic and modular in nature. Given binding peptide and feature data, we expect it can be used to predict other PRM mediated PPIs in yeast and other organisms. Additional features such as network topology, protein expression and text mining derived protein relationships can be added to our framework. Future development includes testing this method on other PRMs in different organisms, especially human.

## Supplementary Material

Supplementary Data
