# Supplementary material for: Predicting physiologically relevant SH3 domain mediated protein–protein interactions in yeast
Source: Bioinformatics. 2016 Feb 9;32(12):1865–72. doi: 10.1093/bioinformatics/btw045 (PMC4908317; doi:10.1093/bioinformatics/btw045)
Supplement: Supplementary Data [file supp_32_12_1865__index.html]

Predicting physiologically relevant SH3 domain mediated protein-protein interactions in yeast — Predicting physiologically relevant SH3 domain mediated protein–protein interactions in yeast — Predicting physiologically relevant SH3 domain mediated protein–protein interactions in yeast — Supplementary Data 

# Predicting physiologically relevant SH3 domain mediated protein–protein interactions in yeast

## Supplementary Data

files

- Supplementary Data - pdf file
