# Supplementary material for: Predicting physiologically relevant SH3 domain mediated protein–protein interactions in yeast
Source: Bioinformatics. 2016 Feb 9;32(12):1865–72. doi: 10.1093/bioinformatics/btw045 (PMC4908317; doi:10.1093/bioinformatics/btw045)
Supplement: Supplementary Data [file supp_btw045_PRM_PPI_supplementary.pdf]

Supplementary Material: Predicting physiologically relevant SH3 domain mediated protein-protein interactions in yeast

Shobhit Jain

December 5, 2015

Position weight matrix and proteome scanning

Position weight matrices (PWMs) are statistical models for representing sequence motifs. They are real valued $m \times n$ matrices, where m is the size of alphabet (20 amino acids for protein sequences) and n is the motif length. PWMs contain a weight for each alphabet symbol i at each position j in the motif. Weight can be described as a log-odds score of a probabilistic model against a background [Pizzi et al., 2011].

$$M(i,j) = \log \frac{P(i,j)}{B(i)} \tag{1}$$

where $B(i)$ is the background probability of amino acid i in the proteome and $P(i,j)$ is the probability of amino acid i at position j.

$$P(i,j) = \frac{count(i,j)}{N} \tag{2}$$

where $count(i,j)$ is the empirical count of amino acid i at position j and N is the count of all the amino acids at position j. Low information content positions or columns at the edges of PWMs are removed to improve signal of the core motif. The information content of each position in the motif is calculated as [Erill, 2012],

$$IC(j) = \left[-\sum_{i=1}^{m} B(i) \log B(i) \right] - \left[-\sum_{i=1}^{m} P(i,j) \log P(i,j) \right] \tag{3}$$

where $IC(j)$ is the mutual information content of jth position in the motif. Information content ratio is then calculated as,

$$ICR(j) = \frac{IC(j)}{IC_{max}} \tag{4}$$

Amino acid positions on both ends of the motif with $ICR(j) \leq 0.4$ are removed. Trimmed PWMs are used to scan a protein sequence to find matches of the weighted pattern above a threshold score (k). For a protein sequence ($S = s_1 s_2 s_3 ...$) the match score ($W(s)$) of any m amino acid long segment is the sum of individual amino acid weights in the PWM [Pizzi et al., 2011].

$$W(s_j..s_{j+m-1}) = \sum_{j=1}^{m} M(s_j, j) \tag{5}$$

where $M(s_i, j)$ is the log-odds score of amino acid s_i at position j in the PWM. The number of statis-

tically significant matches are controlled by converting match score thresholds to p-values. For a given PWM the relationship between its match scores and p-values is defined such that in the background distribution match scores $W(s) \geq k$ [Pizzi et al., 2011, Wu et al., 2000]. Not all amino acid positions within a motif are significant. For example, in class 1 SH3 binding motif [R/K]xxPxxP, positions 1, 4, and 7 are more significant than others. Amino acid positions with $(IC(j)) \geq 0.5$ within the trimmed PWMs are identified as significant. These significant amino acid positions are used in calculation of disordered region, surface accessibility, and peptide conservation scores.

Bayesian integration

The objective of a Bayesian PPI prediction model is to estimate the probability that a given protein pair interacts, conditioned on the biological evidence in support of that interaction. A naïve Bayesian model simplifies this problem by assuming independence between different types of biological evidence. For a protein pair described by a set of features ($X_i = X_1, X_2, ...X_n$) a naïve Bayes PPI prediction model is defined as,

$$
\begin{aligned}
\arg\max_Y P(Y|X_i) &= \arg\max_Y \frac{P(X_i|Y)P(Y)}{P(X_i)} \\
&= \arg\max_Y P(Y) \prod_i P(X_i|Y) \\
\arg\max_Y \log P(Y|X_i) &= \arg\max_Y \log P(Y) + \sum_i \log P(X_i|Y)
\end{aligned} \tag{6}
$$

where $P(Y)$ is the class prior probability and $P(X_i|Y)$ is the class-conditional probability. As there are only two classes $Y \in \{\text{interacting}, \text{non-interacting}\}$ therefore class priors are estimated by treating $P(Y)$ as a multinomial (or categorical) distribution $P(Y) = \Pi_Y$. All continuous peptide and protein features are discretized by binning and modeled using a multinomial probability distribution $P(X_i|Y) = \text{Multi}(X_i; \theta_{iY}) \propto \Theta_{iY}^{X_i}$. Putting it all together, the naïve Bayesian model is defined as,

$$
\arg\max_Y \log P(Y|X_i) = \arg\max_Y \log \Pi_Y + \sum_i \log \Theta_{iY}^{X_i} \tag{7}
$$

where model parameters Π_Y and $\Theta_{iY}^{X_i}$ are learned from the training data set. While modeling the PRM mediated PPI prediction problem a set of observations are made on domain-peptides while others are made on full-length proteins. Assuming that peptide and protein features are independent of each other, two separate naïve Bayes models M_{pep} for peptide features and M_{pro} for protein features are built to independently assess the class probability Y. The posterior probabilities $P(Y|M_{pep})$ and $P(Y|M_{pro})$ are combined using Bayes' theorem [Mitchell, 1997],

$$
P(Y|M_{pep}, M_{pro}) = \frac{P(Y)P(M_{pep}, M_{pro}|Y)}{P(M_{pep}, M_{pro})} \tag{8}
$$

as M_{pep} and M_{pro} are independent therefore, they are conditionally independent given the class Y,

$$P(M_{pep}, M_{pro}|Y) = P(M_{pep}|Y)P(M_{pro}|Y) \tag{9}$$

substituting $P(M_{pep}, M_{pro}|Y)$ in equation (8),

$$P(Y|M_{pep}, M_{pro}) = \frac{P(Y)P(M_{pep}|Y)P(M_{pro}|Y)}{P(M_{pep}, M_{pro})} \tag{10}$$

re-writing $P(M_{pep}|Y)$ and $P(M_{pro}|Y)$ using Bayes theorem,

$$\begin{aligned} P(Y|M_{pep}, M_{pro}) &= \frac{P(Y)P(Y|M_{pep})P(M_{pep})P(Y|M_{pro})P(M_{pro})}{P(Y)P(Y)P(M_{pep}, M_{pro})} \\ &= \frac{P(M_{pep})P(M_{pro})}{P(M_{pep}, M_{pro})} \times \frac{P(Y|M_{pep})P(Y|M_{pro})}{P(Y)} \\ &= \alpha \frac{P(Y|M_{pep})P(Y|M_{pro})}{P(Y)} \end{aligned} \tag{11}$$

$\alpha = \frac{P(M_{pep})P(M_{pro})}{P(M_{pep}, M_{pro})}$ is a class independent term and thus can be treated as normalization constant to ensure $\sum_i P(Y_i|M_{pep}, M_{pro}) = 1$.

Model training

Peptide classifier positive set (P1)

MUSI [Kim et al., 2011] is used to identify multiple binding specificities of the 864 unique peptides (sequence length less than 25 amino acids) belonging to 1238 SH3-peptide PPIs from the MINT database [Licata et al., 2012]. This resulted in three generic PWMs capturing major known SH3 domain binding motif classes RxxPxxP, PxxPxR, and PxxP.

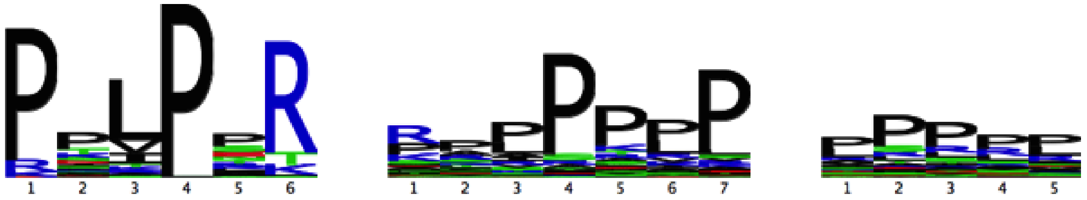

Figure 1: SH3 domain binding motifs in MINT database

All 864 peptides were scored using the three PWMs and only those with scores greater than the stringent p-value threshold of $1e-05$ were retained. This filtering resulted in a set of 683 interactions. Further, interactions with missing feature information are removed thus resulting in a high confidence positive set of 628 SH3 domain-peptide mediated interactions.

Peptide classifier negative set (N1)

The negative dataset consists of randomly selected protein pairs with one member containing a SH3 domain and the other a $10-17$ amino acid long randomly selected proteome sequence. Peptide sequences are scored using positive PWMs from the P1 dataset and only those with scores below the p-value threshold of 0.05 are retained.

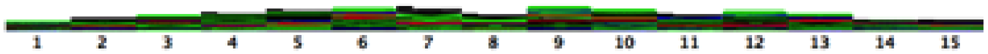

Figure 2: Negative peptide set motif

Also, the protein pairs are not part of known interactions from the iRefIndex (version 13.0) database

[Razick et al., 2008]. Positive (P1) and negative (N1) data sets are balanced with complete feature information.

Protein classifier positive set (P2)

5,795 pairwise yeast PPIs are retrieved from iRefIndex using its web interface iRefWeb [Turner et al., 2010]. iRefIndex consolidates PPIs from 10 major public databases and provides many filters to create a high confidence PPI set. The interactions retrieved from iRefWeb are all physical, experimental, from a single organism, supported by at least two publications and have a MI (MINT-Inspired) score >= 0.5. A high confidence set of 5,215 interactions was created after removing instances with missing protein feature information.

Protein classifier negative set (N2)

5,215 randomly selected protein pairs which are not known yeast interactions (over 117 thousand) from iRefIndex and have complete feature information.

Feature selection

An important assumption behind a naïve Bayesian classifier is that the features are independent of each other. The performance of naïve Bayesian classifier degrades when the involved features are highly correlated [Ratanamahatana and Gunopulos, 2003]. Mutual information is one of the methods for measuring dependence between two variables. Mutual information can capture both linear and non-linear relationships.

$$MI(X,Y) = \sum_{y \in Y} \sum_{x \in X} P(x,y) log \frac{P(x,y)}{P(x)P(y)} \tag{12}$$

where $P(x,y)$ is the joint probability distribution and $P(x)$ and $P(y)$ are the marginal probability distributions. Mutual information score lies within the range $[0,\infty]$. Maximal information coefficient (MIC) technique calculates normalized mutual information scores within the range $[0,1]$ where, a score of 0 indicates complete independence and 1 total dependence between two variables [Albanese et al., 2013, Reshef et al., 2011]. Figure 3 shows the MICs for peptide and protein features. Peptide features: disordered region (DR) and surface accessibility (SA) and protein features: cellular component (CC) and biological process (BP) have MICs of 0.72 and 0.5 respectively.

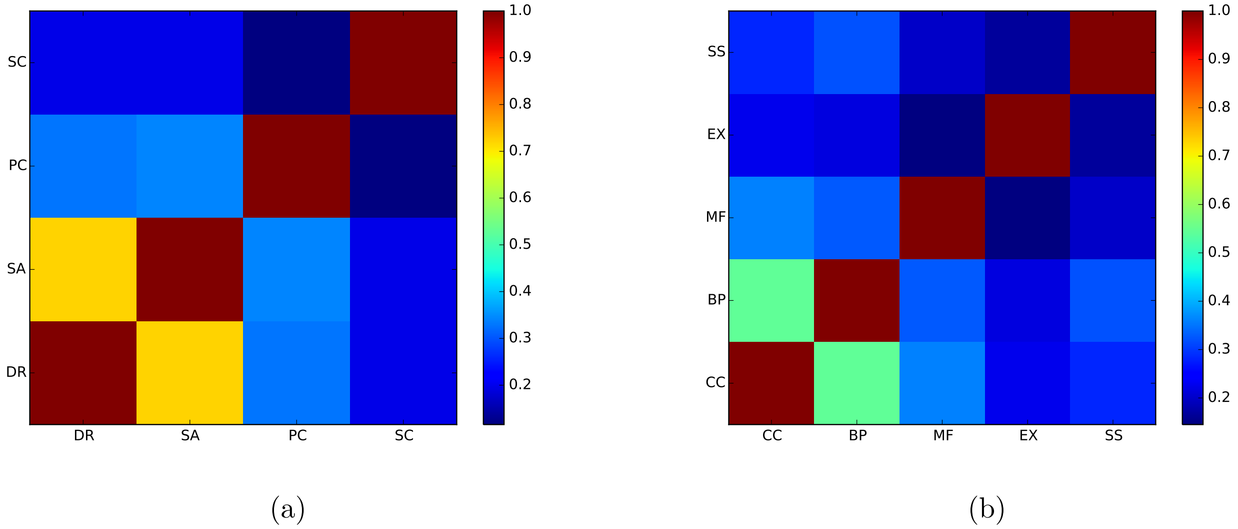

Figure 3: Maximal information coefficients for (a) Peptide feature set: disordered region (DR), surface accessibility (SA), peptide conservation (PC), structural contact (SC). (b) Protein feature set: cellular component (CC), biological process (BP), molecular function (MF), gene expression (EX), sequence signature (SS)

To analyze the effect of correlation between DR and SA in peptide feature set and CC and BP in protein

feature set on the performance of naïve Bayesian classifier we built four different classifiers without one of the correlated features: (-)DR, (-)SA, (-)CC, and (-)BP and compared their performance with classifiers built using all features (ALL) using different statistics. Moreover, to identify the feature subset which maximizes the performance of both classifiers we compared all possible feature combinations. We computed average area under ROC curve (AUROC), area under precision-recall curve (AUPRC), Brier score (BRIER), F_1-score, Matthews correlation coefficient (MCC) and accuracy (ACC) of 10-fold cross-validation protocol to determine the performance of different models. The peptide classifier was trained and tested using P1 & N1 datasets and the protein classifier using P2 & N2. F_1-score, MCC and ACC are reported at threshold score ≥ 0.9. All measures except the Brier score are directly proportional to performance i.e. the higher the score for a model, the better the performance. On the other hand, the lower the Brier score for a model, the better the performance. Except MCC, which lies within the range $[-1, 1]$, other measures are within $[0, 1]$ range. It is clear from the Tables 1 and 2 that removing any of the individual features or any of the combinations do not improve the performance of either classifier. Even removing one of the correlated features does not improve the performance. For the peptide classifier, F_1-score, MCC, and ACC drop sharply for (-)DR and (-)SA models. Similarly, for the protein classifier, the performance degrades when either BP or CC are removed.

Model	AUROC	AUPRC	BRIER	F_1-score	MCC	ACC
ALL	0.94	0.93	0.09	0.86	0.73	0.86
(-) DR	0.93	0.92	0.09	0.64	0.45	0.68
(-) SA	0.94	0.93	0.09	0.65	0.46	0.69
(-) PC	0.92	0.9	0.1	0.84	0.69	0.84
(-) SC	0.92	0.92	0.1	0.87	0.73	0.86
(-) DR, SA	0.78	0.77	0.19	0.47	0.26	0.57
(-) DR, PC	0.91	0.88	0.1	0.69	0.47	0.71
(-) DR, SC	0.92	0.91	0.11	0.54	0.34	0.61
(-) SA, PC	0.93	0.91	0.1	0.72	0.52	0.74
(-) SA, SC	0.92	0.91	0.11	0.55	0.35	0.62
(-) PC, SC	0.9	0.91	0.11	0.86	0.72	0.86
(-) DR, SA, PC	0.72	0.68	0.21	0.33	0.0	0.5
(-) DR, SA, SC	0.64	0.7	0.23	0.48	0.27	0.57
(-) DR, PC, SC	0.88	0.9	0.12	0.33	0.0	0.5
(-) SA, PC, SC	0.88	0.9	0.11	0.33	0.0	0.5

Table 1: Peptide classifier: area under ROC curve (AUROC), area under precision-recall curve (AUPRC), Brier score (BRIER), F_1-score, Matthews correlation coefficient (MCC) and accuracy (ACC) for different models.

Model	AUROC	AUPRC	BRIER	F_1-score	MCC	ACC
ALL	0.98	0.98	0.06	0.9	0.81	0.9
(-) CC	0.97	0.98	0.06	0.89	0.8	0.89
(-) BP	0.97	0.98	0.06	0.89	0.8	0.89
(-) MF	0.97	0.98	0.06	0.9	0.81	0.9
(-) EX	0.97	0.98	0.07	0.89	0.8	0.89
(-) SS	0.95	0.96	0.08	0.88	0.78	0.88
(-) CC, BP	0.96	0.97	0.07	0.84	0.72	0.84
(-) CC, MF	0.97	0.97	0.07	0.88	0.78	0.88
(-) CC, EX	0.97	0.97	0.07	0.87	0.76	0.87
(-) CC, SS	0.94	0.95	0.09	0.85	0.73	0.85
(-) BP, MF	0.97	0.97	0.07	0.88	0.78	0.88
(-) BP, EX	0.97	0.97	0.07	0.86	0.76	0.87
(-) BP, SS	0.93	0.95	0.09	0.86	0.76	0.87
(-) MF, EX	0.97	0.98	0.07	0.88	0.78	0.88
(-) MF, SS	0.94	0.96	0.09	0.87	0.76	0.87
(-) EX, SS	0.93	0.95	0.09	0.86	0.75	0.86
(-) CC, BP, MF	0.94	0.95	0.09	0.79	0.64	0.79
(-) CC, BP, EX	0.94	0.95	0.09	0.82	0.68	0.82
(-) CC, BP, SS	0.88	0.91	0.12	0.81	0.67	0.81
(-) CC, MF, EX	0.96	0.97	0.08	0.84	0.72	0.85
(-) CC, MF, SS	0.93	0.94	0.1	0.82	0.69	0.82
(-) CC, EX, SS	0.91	0.94	0.11	0.79	0.65	0.8
(-) BP, MF, EX	0.96	0.97	0.07	0.84	0.73	0.85
(-) BP, MF, SS	0.91	0.94	0.1	0.85	0.73	0.85
(-) BP, EX, SS	0.91	0.93	0.11	0.84	0.72	0.85
(-) MF, EX, SS	0.92	0.94	0.1	0.85	0.73	0.85
(-) CC, BP, MF, EX	0.9	0.92	0.12	0.69	0.51	0.71
(-) CC, BP, MF, SS	0.8	0.86	0.16	0.72	0.56	0.74
(-) CC, BP, EX, SS	0.77	0.84	0.18	0.66	0.48	0.69
(-) CC, MF, EX, SS	0.9	0.92	0.12	0.77	0.62	0.78
(-) BP, MF, EX, SS	0.87	0.91	0.12	0.8	0.67	0.81

Table 2: Protein classifier: area under ROC curve (AUROC), area under precision-recall curve (AUPRC), Brier score (BRIER), F_1-score, Matthews correlation coefficient (MCC) and accuracy (ACC) for different models.

Figure S1

Change in average area under the curve (AUC) with the number of yeast gene expression datasets used for predicting PPIs. This figure was generated by randomly selecting (repeated 100 times) yeast gene expression datasets in incremental fashion and doing receiver operating characteristic (ROC) analysis.

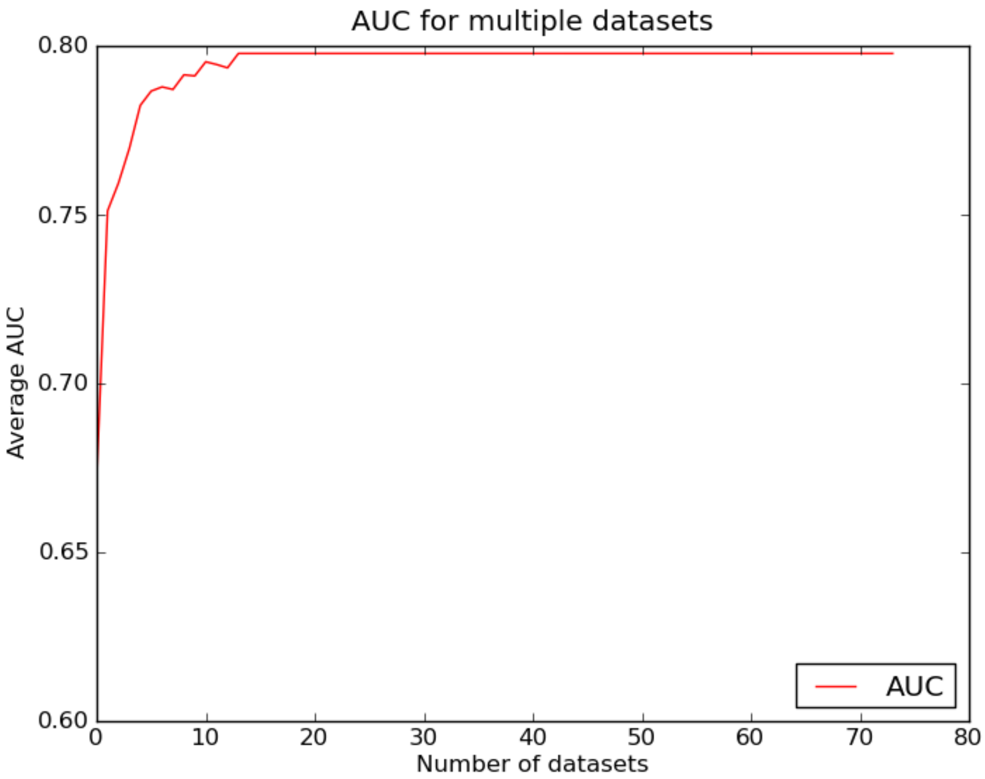

Figure S2

Distribution of positive and negative dataset score for peptide and protein features.

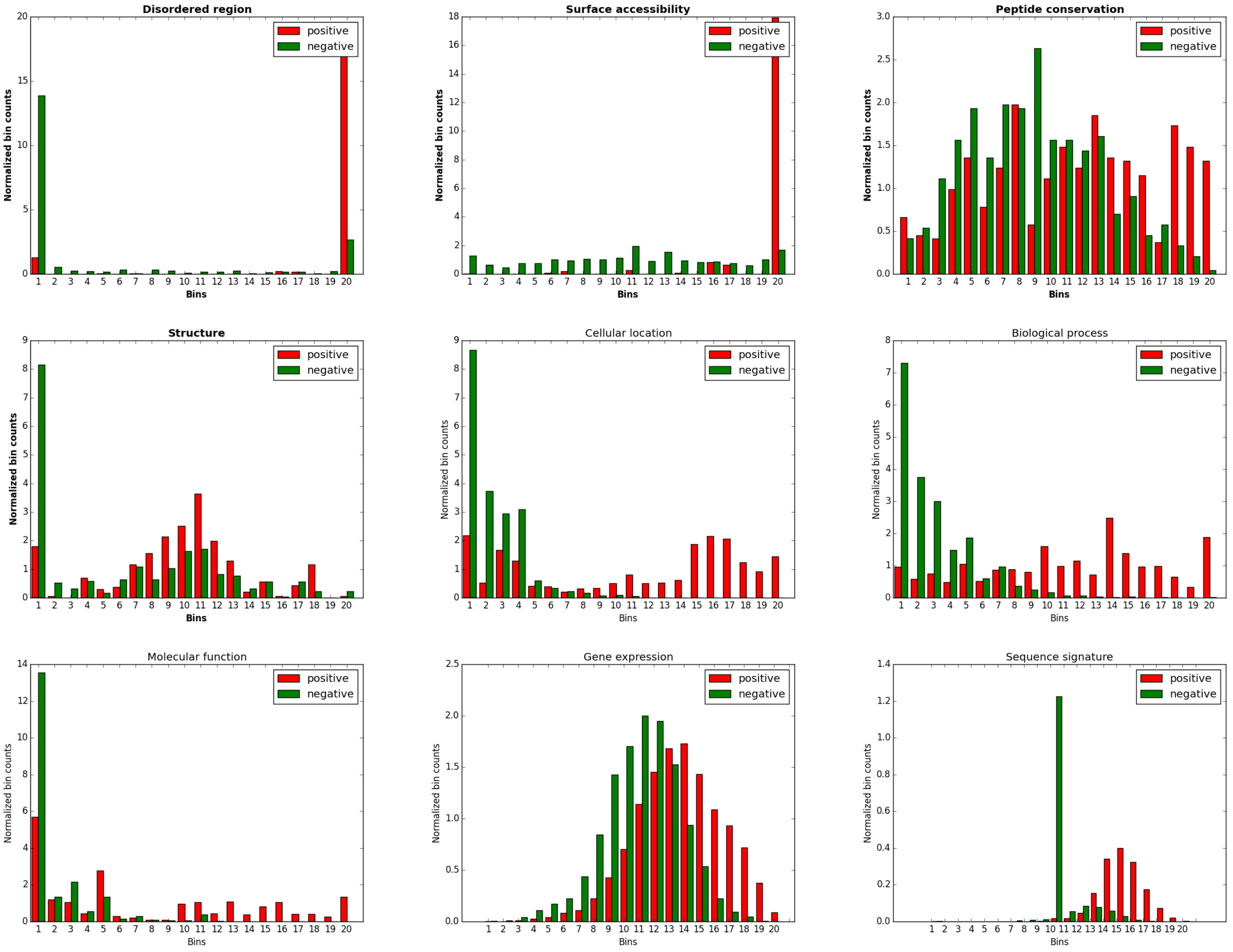

Table S1

List of yeast SH3 domains from Tonikian *et al.* (2009) and their sequences.

Domain id	Domain Sequence
P15891	PWATAEYDYDAAEDNELTFVENDKIINIEFVDDDWWLGELEKDGSKGLFPSNYVSLGN
P47068_classIIcombined	MSEPEVPFKVVAQFPYKSDYEDDLNFEKDQEIIVTSVEDAEWYFGEYQDSNGDVIEGIF-PKSFVAVQGSEVGKEAESS
P29366-1	SQRDSSPKNRHNSKDITSPEKVIKAKYSYQAQTSKELSFMEGEFFYVSGDEKDWYKAS-NPSTGKEGVVPKTYFEVFDRTKPSSVNGS
P29366-2_PXXP	NGSNSSSRKVTNDSLNMGSLYAIVLYDFKAEKADELTTYVGENLFICAHHNCEWFIAKPI-GRLGGPGLVPVGFVSIIDIATGYATGNDV
P38041	MSLEGNTLGKGAKSFPLYIAVNQYSKRMEDELNMKPGDKIKVITDDGEYNDGWYYGRNL-RTKEEGLYPAVFTKRIAIEKPENLHKS
P39969	DSKGSATGRDGGNFPMYIAINEYFKRMEDELDMKPGDKIKVITDDEEYKDGWYFGRNL-RTNEEGLYPVVFTQKITVEKAPTLMRA
P38822-1	RTTSTNNTKKTTQNSSDDGKNKVLYAYVQKDDDEITITPGDKISLVARDTGSGWTKIN-NDTTGETGLVPTTYIRISSAATVKANDRGPAPEVPPP
P38822-2	EVPPPRRSTLPVRTMEAIYAYEAQGDDEISIDPGDIITVIRGDDGSGWTYGECDGLKGLF-PTSYCK
Q07533_classI	MATNLTSLKPPFKVKARYGWSGQTKGDLGFLEGDIMEVTRIAGSWFYGKLLRNKKCS-GYFPHNFVILLEERLNSSTENGRQPS
Q07533_classII	MATNLTSLKPPFKVKARYGWSGQTKGDLGFLEGDIMEVTRIAGSWFYGKLLRNKKCS-GYFPHNFVILLEERLNSSTENGRQPS
P11710	EASVQLGKTYTVIQDYEPRLTDEIRISLGEKVKILATHTDGWCLVEKCNTQKGSIHVSVD-DKRYLNEDRGIVPGDCLQEYD
Q05080	LPIVTSEGFPVIEYAKAMYPLIGNEAPGLANFHKGDYLLITEIVNKDWYKGEVYD-NDRIDRNHRIGLIPYNFIQLLHQGL
P38753	APAHKIPAQTVVRRVRALYDLTTNEPDELSFRKGDVITVLEQVYRDWWKGALRGNMGIF-PLNYVTPIVEPSKEEIEKE
P53281_classI	NQRSPQNADTEEYVEALYDFEAQQDGDLSLKTGDKIQVLEKISPDWYRGKSNNKIGIFPA-NYVKPAFTRSASPKSAEA
P53281_classII	NQRSPQNADTEEYVEALYDFEAQQDGDLSLKTGDKIQVLEKISPDWYRGKSNNKIGIFPA-NYVKPAFTRSASPKSAEA
P43603	PQTSQGRFTAPTSPSTSSPKAVALYSFAGEESGDLPFRKGDVITILKKSDSQNDWWT-GRVNGREGIF
P32793	NESTATNSATPTAVALYNFAGEQPGDLAFKKGDVITILKKSDSQNDWWTGRTNGKEGIF-PANYVRVS
P36006	QPKDPKFEAAYDFPGSGSSSELPLKKGDIVFISRDEPSGWSLAKLLDGSKEGWVPTAYMT-PYKDTRNTVPVAATGAV
Q04439	IPPPPPPPPPSSKPKEPMFEAAYDFPGSGSPSELPLKKGDVIYITREEPSGWSLGK-LLDGSKEGWVPTAYMKPHSGNNNIPTPPQNRDV

Q12163_PXXP	ITLPDDYIVNQRAVALYDFEPENDNELRLAEGDIVFISYKHGQGWLVAENESGSKT-GLVPEEFVSYIQPEDGENEVEN
P80667_classIIA	SQGNGSEPIDPSKLEFARALYDFVPENPEMEVALKKGDLMAILSKKDPLGRDSD-WWKVRTKNGNIGYIPYNYIEIIKRRKKIEHVDDETRTH
P80667_classIIB	SQGNGSEPIDPSKLEFARALYDFVPENPEMEVALKKGDLMAILSKKDPLGRDSD-WWKVRTKNGNIGYIPYNYIEIIKRRKKIEHVDDETRTH
Q06449_classI	PASLEYVEALYQFDPQQDGDLGLKPGDKVQLLEKLSPEWYKGSCNGRTGIFPANYVK-PAFSGSNGPSNLP
Q06449_classII	PASLEYVEALYQFDPQQDGDLGLKPGDKVQLLEKLSPEWYKGSCNGRTGIFPANYVK-PAFSGSNGPSNLP
P39743_ClassI	AAPGVETVTALYDYQAQAAGDLSFPAGAVIEIVQRTPDVNEWWTGRYNGQQGVF-PGNYVQLNKN
P39743_ClassII	AAPGVETVTALYDYQAQAAGDLSFPAGAVIEIVQRTPDVNEWWTGRYNGQQGVF-PGNYVQLNKN
P40073	GDTLGLYSDIGDDNFIYKAKALYPYDADDDDAYEISFEQNEILQVSDIEGRWWKAR-RANGETGIIPSNYVQLIDGPEEMHR
P32790-1_classI	MTVFLGIYRAVYAYEPQTPEELAIQEDDLLYLLQKSDIDDWWTVKKRVIGSDSEEP-VGLVPSTYIEEAPVLKKVRAIYD
P32790-2_classII	VPSTYIEEAPVLKKVRAIYDYEQVQNADEELTFHENDVFDVFDDKDADWLLVKSTVSNE-FGFIPGNYVEPENGSTSKQEQA
P32790-3	GLREVEMASKSKKRGIVQYDFMAESQDELTIKSGDKVYILDDKKSKDWWMCQLVDSGKS-GLVPAQFIEPVRDKKHTESTAS

Table S2

List of yeast SH3 domains from Tonikian *et al.* (2009) and their binding motifs (trimmed) with significant amino acid positions within those motifs.

Domain id	Phage logo	Significant positions
P11710	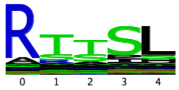	0, 1, 2, 3, 4
P15891	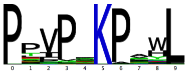	0, 2, 3, 5, 6, 8, 9
P29366-1	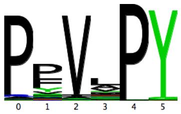	0, 2, 4, 5
P29366-2_PXXP	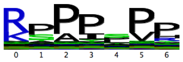	0, 1, 2, 3, 5, 6
P32790-1_classI	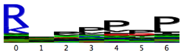	0, 4, 6
P32790-2_classII	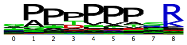	0, 1, 2, 3, 4, 5, 6, 8
P32790-3	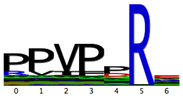	0, 1, 2, 3, 5
P32793	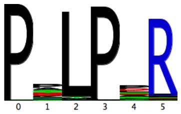	0, 2, 3, 5
P36006	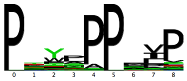	0, 4, 5, 8
P38041	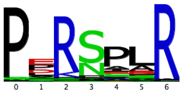	0, 2, 3, 4, 5, 6
P38753	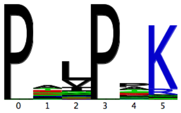	0, 3, 5
P38822-1	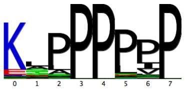	0, 2, 3, 4, 5, 6, 7
P38822-2	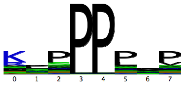	3, 4
P39743_ClassI	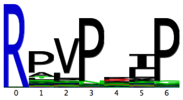	0, 1, 2, 3, 6

P39743_ClassII	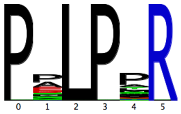	0, 2, 3, 5
P39969	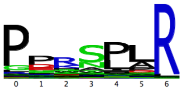	0, 3, 4, 5, 6
P40073	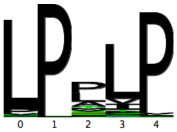	0, 1, 3, 4
P43603	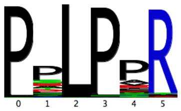	0, 2, 3, 5
P47068_classIIcombined	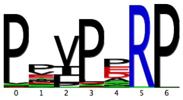	0, 2, 3, 5, 6
P53281_classI	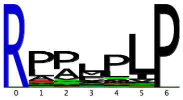	0, 1, 5, 6
P53281_classII	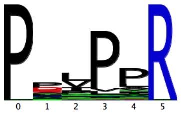	0, 3, 5
P80667_classIIA	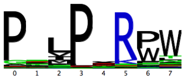	0, 2, 3, 5, 6, 7
P80667_classIIB	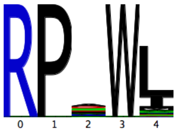	0, 1, 3, 4
Q04439	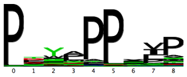	0, 4, 5, 8
Q05080	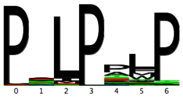	0, 2, 3, 6
Q06449_classI	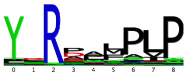	0, 2, 6, 7, 8
Q06449_classII	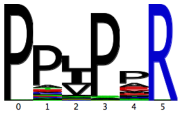	0, 1, 2, 3, 5
Q07533_classI	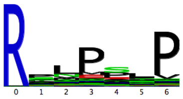	0, 3, 6
Q07533_classII	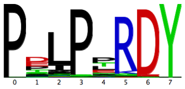	0, 2, 3, 5, 6, 7
Q12163_PXXP	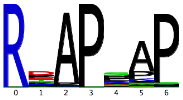	0, 2, 3, 5, 6

Table S3

SH3 domain mediated PPIs in yeast.

Download link for predictions: DoMo-Pred

Table S4

Enrichment analysis of predicted high confidence interactors.

P-value	Term ID	Term name	Proteins
0.00113	KEGG:04011	MAPK signaling pathway - yeast	P24583, P32917, Q03497, P08018, P41832, P32491
0.0375	KEGG:04144	Endocytosis	P34216, P25604, P35197, P40343, Q12446
0.00077	KEGG:04070	Phosphatidylinositol signaling system	P24583, P34756, P50942, Q12271
0.0169	KEGG:00562	Inositol phosphate metabolism	P34756, P50942, Q12271
0.00698	REAC:5733237	Innate Immune System	Q03306, Q03497, P08018, Q12236, Q12446, P32491
0.00316	REAC:5733336	Fc epsilon receptor (FCERI) signaling	Q03306, P08018, Q12236, P32491
0.00000197	REAC:5733138	Signal Transduction	P24583, Q03306, Q04739, Q03497, P40450, P32521, P41832, Q12236, Q12446, P48582, P32873, P32491
2.97E-09	REAC:5733143	Signaling by Rho GTPases	P24583, Q03306, Q03497, P40450, P32521, P41832, Q12236, Q12446, P48582, P32873
1.65E-08	REAC:5733142	RHO GTPase Effectors	P24583, Q03306, Q03497, P40450, P41832, Q12236, Q12446, P48582
0.05	REAC:5733141	RHO GTPases activate PKNs	P24583, Q03306, Q12236
0.0337	REAC:5733628	Signaling by ERBB4	Q03306, Q12236, P32491
0.0337	REAC:5733629	Signaling by SCF-KIT	Q03306, Q12236, P32491
0.0314	REAC:5733228	Signalling by NGF	Q03306, P32521, Q12236, P32873, P32491
0.0123	REAC:5733461	Costimulation by the CD28 family	Q03306, Q03497, Q12236
0.0123	REAC:5733460	CD28 co-stimulation	Q03306, Q03497, Q12236

Table S5

Enrichment analysis of predicted MYO3 interactors.

P-value	Term ID	Term name	Proteins
0.04	REAC:5733141	RHO GTPases activate PKNs	Q03306, Q12236
0.000817	REAC:5733234	Signaling by ERBB2	Q03306, Q12236, P32491
0.000817	REAC:5733232	Signaling by EGFR	Q03306, Q12236, P32491
0.000817	REAC:5733230	Signaling by PDGF	Q03306, Q12236, P32491
0.000161	REAC:5733628	Signaling by ERBB4	Q03306, Q12236, P32491
0.000344	REAC:5733311	VEGFA-VEGFR2 Pathway	Q03306, Q12236, P32491
0.00337	REAC:5733625	PIP3 activates AKT signaling	Q03306, Q12236
0.000473	REAC:5733336	Fc epsilon receptor (FCERI) signaling	Q03306, Q12236, P32491
0.00376	REAC:5733190	IGF1R signaling cascade	Q03306, Q12236, P32491
0.00337	REAC:5733185	Activation of AKT2	Q03306, Q12236
0.000817	REAC:5733242	Signaling by FGFR	Q03306, Q12236, P32491
0.00337	REAC:5733405	Downstream TCR signaling	Q03306, Q12236
0.00337	REAC:5733635	CD28 dependent PI3K/Akt signaling	Q03306, Q12236
0.000161	REAC:5733629	Signaling by SCF-KIT	Q03306, Q12236, P32491
0.00376	REAC:5733187	IRS-mediated signalling	Q03306, Q12236, P32491

References

Cinzia Pizzi, Pasi Rastas, and Esko Ukkonen. Finding significant matches of position weight matrices in linear time. *Computational Biology and Bioinformatics, IEEE/ACM Transactions on*, 8(1):69–79, 2011.

Ivan Erill. Information theory and biological sequences: Insights from an evolutionary perspective. *Information Theory: New Research. New York: Nova Science Publishers*, pages 1–28, 2012.

Thomas D Wu, Craig G Nevill-Manning, and Douglas L Brutlag. Fast probabilistic analysis of sequence function using scoring matrices. *Bioinformatics*, 16(3):233–244, 2000.

Tom M. Mitchell. *Machine Learning*. McGraw-Hill, New York, 1997.

TaeHyung Kim, Marc S Tyndel, Haiming Huang, Sachdev S Sidhu, Gary D Bader, David Gfeller, and Philip M Kim. MUSI: an integrated system for identifying multiple specificity from very large peptide or nucleic acid data sets. *Nucleic acids research*, page gkr1294, 2011.

Luana Licata, Leonardo Briganti, Daniele Peluso, Livia Perfetto, Marta Iannuccelli, Eugenia Galeota, Francesca Sacco, Anita Palma, Aurelio Pio Nardozza, Elena Santonico, Luisa Castagnoli, and Gianni Cesareni. MINT, the molecular interaction database: 2012 update. *Nucleic Acids Res*, 40(Database issue):D857–D861, Jan 2012. doi: 10.1093/nar/gkr930. URL `http://dx.doi.org/10.1093/nar/gkr930`.

Sabry Razick, George Magklaras, and Ian M Donaldson. iRefIndex: a consolidated protein interaction database with provenance. *BMC Bioinformatics*, 9(1):405, 2008.

Brian Turner, Sabry Razick, Andrei L. Turinsky, James Vlasblom, Edgard K. Crowdy, Emerson Cho, Kyle Morrison, Ian M. Donaldson, and Shoshana J. Wodak. iRefWeb: interactive analysis of consolidated protein interaction data and their supporting evidence. *Database (Oxford)*, 2010:baq023, 2010. doi: 10.1093/database/baq023. URL `http://dx.doi.org/10.1093/database/baq023`.

Chotirat" ann" Ratanamahatana and Dimitrios Gunopulos. Feature selection for the naive bayesian classifier using decision trees. *Applied Artificial Intelligence*, 17(5-6):475–487, 2003.

Davide Albanese, Michele Filosi, Roberto Visintainer, Samantha Riccadonna, Giuseppe Jurman, and Cesare Furlanello. minerva and minepy: a c engine for the mine suite and its r, python and matlab wrappers. *Bioinformatics*, 29(3):407–408, 2013.

David N Reshef, Yakir A Reshef, Hilary K Finucane, Sharon R Grossman, Gilean McVean, Peter J Turnbaugh, Eric S Lander, Michael Mitzenmacher, and Pardis C Sabeti. Detecting novel associations in large data sets. *Science*, 334(6062):1518–1524, 2011.
